# A 1‐year forensic evaluation of DNA degradation and STR typing in embalmed human tissues: Muscle, brain, liver, and bone marrow

**DOI:** 10.1111/1556-4029.70345

**Published:** 2026-04-24

**Authors:** Alyssa Venditti, Mark Luciano, Sophia Tucker, Stacey Sainte‐Marie, Guy Sovak, Aaron Teitelbaum, C. Sean Bohun, Hélène N. LeBlanc

**Affiliations:** ^1^ Faculty of Science Ontario Tech University Oshawa Ontario Canada; ^2^ The Canadian Memorial Chiropractic College (CMCC) Toronto Ontario Canada

**Keywords:** DNA degradation, embalmed cadavers, forensic DNA, postmortem body preservation, STR loci, tissue samples

## Abstract

Postmortem perfusion is a procedure which provides in‐vivo fixation of the human body and prevents organ and tissue decomposition after biological death occurs. Formaldehyde‐based embalming solutions influence nucleic acid degradation, which reduces the quality and quantity of DNA extracted and the effectiveness of short tandem repeat (STR) typing. This research is a 1‐year study aimed at determining the timeframe within which viable DNA profiles can be generated from different tissue types, post‐embalming. Samples from the bone marrow (tibia), trapezius and quadriceps muscles, liver, and brain tissues were collected before embalming and at seven time points post‐embalming. DNA extraction and quantification were performed on each sample to assess whether sufficient DNA was available for testing. Samples with DNA quantities over 0.05 ng/μL underwent amplification and STR analysis to produce DNA profiles. Quantification values and profile quality were compared across tissue types and time points. Results showed that prolonged exposure to embalming solutions significantly reduced DNA quality, leading to less discernible profiles. Statistically significant differences were detected among all tissue types at all time periods. Liver and brain samples retained more DNA than bone marrow, trapezius, and quadricep muscle samples. Qualitative evaluation of ski slope patterns in liver STR profiles demonstrated increasing signal degradation over time and was consistent across loci, suggesting predictable fragment length‐dependent decline. This study suggests that after embalming, the liver is the preferential tissue for recovering DNA for forensic analysis, as the liver samples were the most robust and the only sample type to yield DNA profiles up to 1 year.

**Highlights**

- First assessment of DNA STR success rates across multiple tissue types over a 1‐year period.
- Liver was the preferred tissue for post‐embalming DNA recovery; the only sample to yield amplifiable DNA at 1 year.
- A qualitative ski slope analysis of liver, showing reduced peak height as that amplicon size increased.
- First published use of blood samples prior to embalming as control samples.

## 1 INTRODUCTION

The process of decomposition begins almost immediately after death [1, 2, 3]. Embalming is a method of preserving the body of the deceased by perfusing it with an array of chemicals [4]. This treatment stops the process of putrefaction, allowing the structure and appearance of the body to be maintained for a few months and up to several years [5]. The prolonged preservation of the human body is beneficial to various fields, including the funeral industry, academia, and research [6]. The first step in the cadaveric embalming is the creation of a decompression opening in the occipital bone in the midline immediately above the superior nuchal line, and an opening in the sagittal sinus, which aids in the fixation of the brain by allowing the embalming solution to perfuse inside the skull [7]. The embalming solution is delivered into the body via the femoral artery access located in the femoral triangle [8]. The volume of the embalming solution pumped into the body during embalming is determined by the size and condition of the body [8, 9]. After the body is perfused with the embalming solution, it is left to fix for at least 7 days. At this point, the Infutrace™ solution is introduced overnight, which reduces the toxic vapor levels of formaldehyde and phenol used in the embalming process [7].

The embalming process accelerates the degradation of DNA. The chemicals in the embalming solution aid in the preservation of decaying tissue by permeating into the cells, which increases DNA degradation in tissues after prolonged exposure [10, 11]. However, the type of tissue can also affect the progression of DNA degradation. DNA yield, extent of degradation, and the ability to generate a short tandem repeat (STR) profile correlate with the length of time an embalmed cadaver is stored before tissue is collected. Therefore, increased cadaveric storage times lead to increased degradation of DNA and a decrease in DNA yield [12, 13, 14]. Todorvic et al. [15] found that in formalin‐fixed brain, lung, and kidney tissues collected during forensic autopsies, both incubation time and storage temperature influenced DNA quality. Although multiple factors contribute to DNA degradation and reduced yields from embalmed tissues, viable DNA can still be recovered. Biological samples play a crucial role in fields such as genetic research and forensic science investigations [16, 17]. The recovery of viable DNA from embalmed tissue has both scientific and legal ramifications. It enables genetic confirmation in cases involving potential misidentification. These include, but are not limited to, historical record discrepancies, disputed identity claims, and facilitate paternity or kinship testing. This also supports forensic investigations for genetic comparison in cold cases or delayed investigations that involve exhumed, embalmed remains. [15, 18, 19].

The current body of research lacks studies comparing DNA degradation across multiple tissue types following embalming [20, 21]. To address this gap, the authors selected five different tissue types for examination in the current study. To investigate whether proximity to the perfusion site influences DNA degradation, samples were taken from tissues adjacent (quadriceps femoris muscle) and remote (trapezius muscle) from the perfusion site [12]. Additional gaps in the literature, such as the lack of replication at sample sites [12, 20], limited variation in collection time points [12, 21], and the absence of pre‐embalming controls [12, 20], were addressed in this study by extracting three tissue replicates per time point, utilizing pre‐embalming control samples and designated sampling time intervals.

The objectives of this research were to assess DNA degradation and the potential to generate interpretable STR profiles following embalming by implementing pre‐embalming controls and sampling at planned time intervals. The effects of embalming were measured on different types of tissue to ultimately determine the most reliable sampling location for DNA up to 1‐year post‐mortem.

## 2 MATERIALS AND METHODS

### 2.1 Research ethics approval

Ethical approval was obtained by the Office of the Chief Coroner for Ontario, the Research Ethics Boards (REBs) of Ontario Tech University (Oshawa, ON), and the Canadian Memorial Chiropractic College (CMCC). The CMCC in Toronto is one of 10 educational institutions under the authority of the Anatomy Act of Ontario, Canada, which allows human cadaveric donors to be delivered to an educational institution for the sole purpose of anatomical dissection and ensures all research remains in compliance with Ontario's Anatomy Act, R.S.O. 1990, c. A.21 (“Act”) [22].

### 2.2 Sampling

A sampling trial was conducted at the CMCC Anatomy Department in Toronto, Ontario, to identify the most appropriate sample types and sampling methods. The factors considered included pre‐existing cadaveric deterioration, collecting enough tissue for successful DNA extraction, and choosing various tissue types. Based on the trial results, the collection sites were selected as the quadriceps femoris (rectus femoris head), liver, trapezius (middle portion), tibial bone marrow, and parietal lobe of the brain. Other locations were excluded due to a higher risk of contamination. The tibia was chosen over other bone marrow sites, such as the femur, because it allows for a cleaner dissection and reduces the risk of cross‐contamination with peripheral blood, which could be more problematic in a femur dissection [23].

### 2.3 Cadaver

A donor was accepted at the CMCC through the Anatomy Act of Ontario. The donor was a 96‐year‐old female, received on February 12, 2023 by the CMCC c. 2 days postmortem from chronic renal failure. The formalin‐based embalming solution required for perfusing the body was produced in batches of 24 liters. Each 24 L of embalming solution, containing: 3 L of 37% formaldehyde solution stabilized in 15% methanol, 1 L of 90% phenol solution, 1 L of 4.8% Dettol (4‐chloro‐3,5‐xylenol), 4 L of 100% ethylene‐glycol (ethane−1,2‐diol) solution, 20 g of thymol (2‐sopropyl‐5‐methylphenol), 13 L of alcohol solution composed of 85% ethanol and 15% methanol, and 2 L of distilled water (Table 1).

**TABLE 1:** Reagents in formalin‐based embalming solution.

Reagent	Volume/mass	Concentration	Manufacturer
Formaldehyde	3 L (stabilized in 15% methanol)	37%	Millipore Sigma
Phenol	1 L	90%	Termo Fisher Scientific
Dettol (4‐chloro‐3,5‐xylenol)	1 L	4.8%	Rb Health (Canada) Inc
Ethylene Glycol (ethane‐1,2‐diol)	4 L	100%	Termo Fisher Scientific
Thymol (5‐methyl‐2‐(propan‐2‐yl)phenol)	20 g	Crystalls	Thermo Scientific Chemicals
Denatured ethanol	13 L	85% ethanol 15% methanol	
Distilled water	2 L		

The donor was stored within a 5°C refrigerator located at the CMCC. Cadaveric perfusion was performed via the right femoral artery on February 13 and 14, 2023 using a Dodge Embalming Machine with 20–40 psi pressure and a flow rate of 650–850 mL/h. A reperfusion was required 16 days after the initial perfusion based on the fluid loss from the cadaver and the state of tissue fixation.

### 2.4 Tissue sampling

Prior to embalming, a longitudinal incision of the femoral artery was made using a scalpel, and six blood samples were collected using sterile swabs. The swabs, stored in EDTA‐coated tubes, served as pre‐embalming controls to provide reference DNA profiles for comparison [23]. Tissue samples were collected from each location, including the quadriceps (rectus femoris), bone marrow (tibia), brain (parietal lobe), liver, and trapezius (middle portion), and each sampling site was sampled at eight sampling time points (Table 2). For the exact sampling method, refer to supplemental information Appendix S1.

**TABLE 2:** Eight sampling time points spanning the duration of 24‐h pre‐embalming to 1‐year post‐embalming.

Sample #	Sampling time point	Date	Days elapsed since initial perfusion
1	24‐h pre‐embalming	February 12, 2023	−1
	Initial perfusion	February 13 and February 14, 2023	0–1
2	24‐h post‐embalming	February 15, 2023	2
3	2‐week post‐embalming	March 1, 2023	16
	Reperfusion	March 2, 2023	17
4	1‐month post‐embalming	March 15, 2023	30
5	2‐month post‐embalming	April 12, 2023	58
6	3‐month post‐embalming	May 11, 2023	88
7	6‐month post‐embalming	August 10, 2023	178
8	1‐year post‐embalming	February 8, 2024	360

A total of 200 tissue samples and 6 blood samples were taken. The tissue samples consisted of five replicates of ~25 mg, removed from the five sampling sites at eight different time points. The amount of tissue removed (~25 mg) was based on the DNA extraction protocols for the DNeasy Blood & Tissue Kit (QIAGEN) [24] and QIAamp DNA Blood Mini Kit (QIAGEN) protocols [25]. All tissue samples were stored in a freezer at −80°C within the Immunology and Cell Biology Laboratory at the CMCC until further processing. For each of the five tissue samples collected per time point, three replicates were extracted for analysis, while the remaining two were stored at −80°C in case additional material was required.

### 2.5 DNA extraction and quantification

DNA extractions (*N* = 123, three aliquots of c. 25 mg from five tissue samples at eight‐time points (120) and three blood samples (3)) were performed at the CMCC in the Immunology & Cell Biology Laboratory using a DNeasy® Blood & Tissue Kit (QIAGEN) for the trapezius, quadriceps, liver, brain, and blood [24], and QIAamp® DNA Blood Mini Kit (QIAGEN) for the bone marrow (tibia) [25].

DNA was quantified at Ontario Tech University using the Applied Biosystems (ABI) Quantifiler™ Duo DNA Quantification Kit (Thermo Fisher Scientific) [26]. Samples were run on the ABI7500 Real Time PCR System (Thermo Fisher Scientific) using the HID Real Time PCR Analysis Software, version 1.3 (Thermo Fisher Scientific). To ensure proper functioning of reagents, a negative and positive control, along with the three extraction blanks, were also run alongside 63 extracted samples.

### 2.6 Amplification and STR analysis

Samples containing sufficient DNA (0.05 ng/μL) were amplified at Ontario Tech University using the AmpFℓSTR™ Identifiler™ Plus PCR Amplification Kit (Thermo Fisher Scientific), followed by amplification on the ABIGeneAmp® PCR System 9700 thermal cycler (Thermo Fisher Scientific).

Amplicons were separated and detected using the ABI SeqStudio Genetic Analyzer (Thermo Fisher Scientific), and subsequent analysis was performed with the Genemapper IDX version 1.6 software (Thermo Fisher Scientific). Fragment analysis was performed using the GS500 LIZ size standard and G5(DS‐33) TM dye set [27].

### 2.7 Statistics

For each of the eight time samples, a Shapiro–Wilk test did not show significant deviation from normality. A single‐factor ANOVA was performed to determine if there exists a statistically significant difference between the locations. If a difference was detected, then Tukey's procedure (α = 0.05) was used to determine the hierarchy of the means. Subsequently, the amount of DNA relative to its pre‐embalming level at each location was statistically determined for the seven time samples after the initial time period. The variance of this was determined assuming no evidence (α = 0.05) of a correlation between the initial and subsequent samples at each of the five locations. The standard error for the ratios is simplified if the underlying data are correlated (ρ ≠ 0) (If X ~ N(μ_x_, σ_x_^2^) and Y ~ N(μ_y_, σ_y_^2^) with cor(X,Y) ≠ 0 then log_e_(Y/X) ~ log_e_(μ_y_/μ_x_) + N(0, σ_x_^2^/μ_x_^2^ + σ_y_^2^/μ_y_^2^)) [27]. This corresponds to a point estimate of the correlation of |*r*| ≥ 0.99692 (Table 5). Ski slope analysis was performed using selected loci from STR profiles of the liver samples.

## 3 RESULTS

This study demonstrated an overall decline of DNA quantity and quality over time for all samples collected, regardless of the tissue type. Results revealed a decrease in DNA quantity in samples as follows: bone marrow (tibia) samples decrease from 3.24 to 0.0018 ng/μL, trapezius samples decreased from 29.42 to 0.0087 ng/μL, quadriceps (rectus femoris) samples decreased from 46.24 to 0.00055 ng/μL, liver samples decreased from 91.74 to 0.3983 ng/μL, and brain samples decreased from 15.15 to 0.0131 ng/μL (Tables 3 and 4). Comparing DNA quality and quantity, pre‐ and 24 h post‐embalming, degradation was observed in all samples, except the brain sample. The brain is distinguished by an increase at the 24 h post‐perfusion sample time.

**TABLE 3:** Map of quantification values (ng/μL) of each extracted sample.

	24‐h pre	24‐h post	2‐week post	1‐month post	2‐month post	3‐month post	6‐month post	1‐year post
Bone marrow (Tibia) 1	3.9465	0.3467	0.0066	0.0249	0.0083	0.0196	0.0090	0.0000
Bone marrow (Tibia) 2	2.6801	0.1012	0.0092	0.0131	0.0062	0.0253	0.0107	0.0053
Bone marrow (Tibia) 3	3.1000	0.2250	0.0063	0.0151	0.0065	0.0183	0.0033	0.0000
Trapezius 1	28.8504	1.3739	1.6683	1.6252	0.0333	0.0165	0.0103	0.0099
Trapezius 2	25.3693	0.9959	2.0027	2.1387	0.0426	0.0109	0.0136	0.0109
Trapezius 3	34.0265	0.9660	1.7589	3.5746	0.0573	0.0092	0.0031	0.0052
Quadriceps 1	40.7636	2.1660	0.6850	1.2243	0.0370	0.0000	0.0032	0.0000
Quadriceps 2	46.2053	2.2332	1.1706	0.4866	0.0317	0.0047	0.0000	0.0164
Quadriceps 3	51.7487	0.8794	0.7679	0.9878	0.0299	0.0213	0.0000	0.0000
Liver 1	93.3165	28.0073	29.1868	12.8482	2.5877	3.0761	1.1645	0.4164
Liver 2	82.5696	25.1673	26.0110	14.9210	1.8897	2.6042	0.5652	0.4184
Liver 3	99.3237	29.1193	30.3317	13.0412	2.2916	2.0269	0.7806	0.3600
Brain 1	11.5123	34.3156	7.3733	9.4178	2.3233	0.4093	0.0817	0.0141
Brain 2	11.2292	22.8770	5.7708	11.0942	2.2020	0.4345	0.1657	0.0158
Brain 3	22.6944	27.1444	5.2214	12.4883	1.5832	0.7496	0.1513	0.0095

*Note*: Red values (<0.05) indicate samples with insufficient DNA for amplification, as per the kit instructions [26, 27]. Yellow values (0.05–0.6) are samples that were not diluted before amplification, and green values (>0.6) are samples that were diluted for amplification.

**TABLE 4:** The *F*_obs_, corresponding *p*‐values based on a distribution of *F*_4,10_, the critical differences used in Tukey's procedure (α = 0.05), and the sample means at each location.

Sample time	24‐h pre	24‐h post	2‐weeks post	1‐month post	2‐month post	3‐month post	6‐month post	1‐year post
*F*_obs_	108.51	84.34	339.55	121.25	72.32	58.95	20.81	376.74
*p* values	3.4 × 10^−8^	1.2 × 10^−7^	1.2 × 10^−10^	2.0 × 10^−8^	2.4 × 10^−7^	6.5 × 10^−7^	7.7 × 10^−5^	7.4 × 10^−11^
Critical diff.	15.3928	7.4221	3.0275	2.6408	0.6358	0.6709	0.3685	0.0419
Means (bone marrow)	3.2422	0.2243	0.0074	0.0177	0.0070	0.0211	0.0077	0.0018
Means (trapezius)	29.4154	1.1119	1.8100	2.4462	0.0444	0.0122	0.0090	0.0087
Means (quadricep)	46.2392	1.7595	0.8745	0.8996	0.0329	0.0087	0.0011	0.0055
Means (liver)	91.7366	27.4313	28.5098	13.6035	2.2563	2.5691	0.8368	0.3983
Means (brain)	15.1453	28.1123	6.1218	11.0001	2.0362	0.5311	0.1329	0.0131

All sample types, except liver, eventually reached a point below the lower threshold (0.05 ng/μL). Each repetition of the liver sample contained sufficient DNA to produce a reliable DNA profile at all sampling time points, spanning 24 h pre‐embalming to 1‐year post‐embalming. The liver also had the largest quantity of DNA across all sampling time periods, with the exception of 24 h post‐embalming, in which the brain samples contained the highest quantity of DNA (Tables 3 and 4).

**FIGURE 1:**
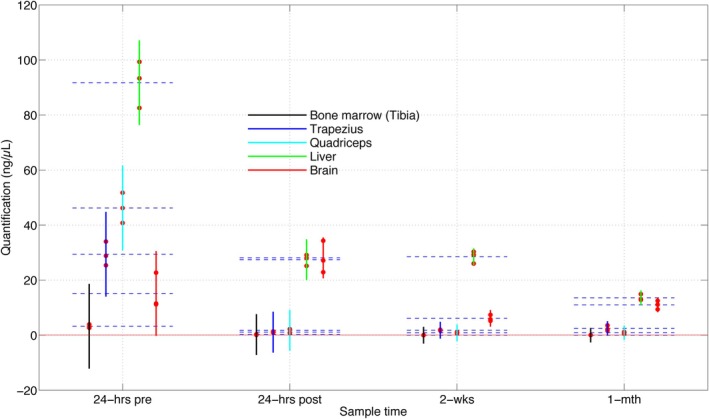
Depiction of the quantification values for the *first* four sample times. Indicated at each epoch are the sample mean (horizontal, blue‐dashed line) and the critical difference (vertical line). The treatments are color coded and are shown in order from left to right, corresponding to bone marrow (tibia), trapezius, quadricep, liver, and brain.

**FIGURE 2:**
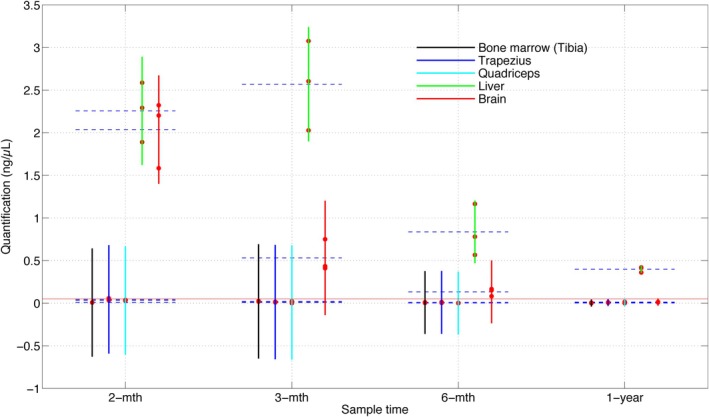
Depiction of the quantification values for the *last* four sample times. Indicated at each epoch are the sample mean (horizontal, blue‐dashed line) and the critical difference (vertical line). The treatments are color‐coded, shown in order from left to right, corresponding to bone marrow (tibia), trapezius, quadriceps, liver, and brain.

A summary of the comparison of the means and the corresponding underlying data is shown in Figures 1 and 2. At all time samples, there exists a significant difference among the locations. The liver and brain tissues consistently yielded higher DNA quantities than the bone marrow (tibia), trapezius, and quadriceps locations, these latter sites being comparatively similar. When all five locations are included, a detectable difference (α = 0.05) persists throughout the year. The overall trend of the hierarchy of the means is

$$\mu \left(\text{bone marrow}\right)<\mu \left(\text{trapezius}\right)<\mu \left(\text{quadriceps}\right)<\mu \left(\text{brain}\right)<\mu \left(\text{liver}\right)$$

The summary statistics for this analysis are presented in Table 4.

**FIGURE 3:**
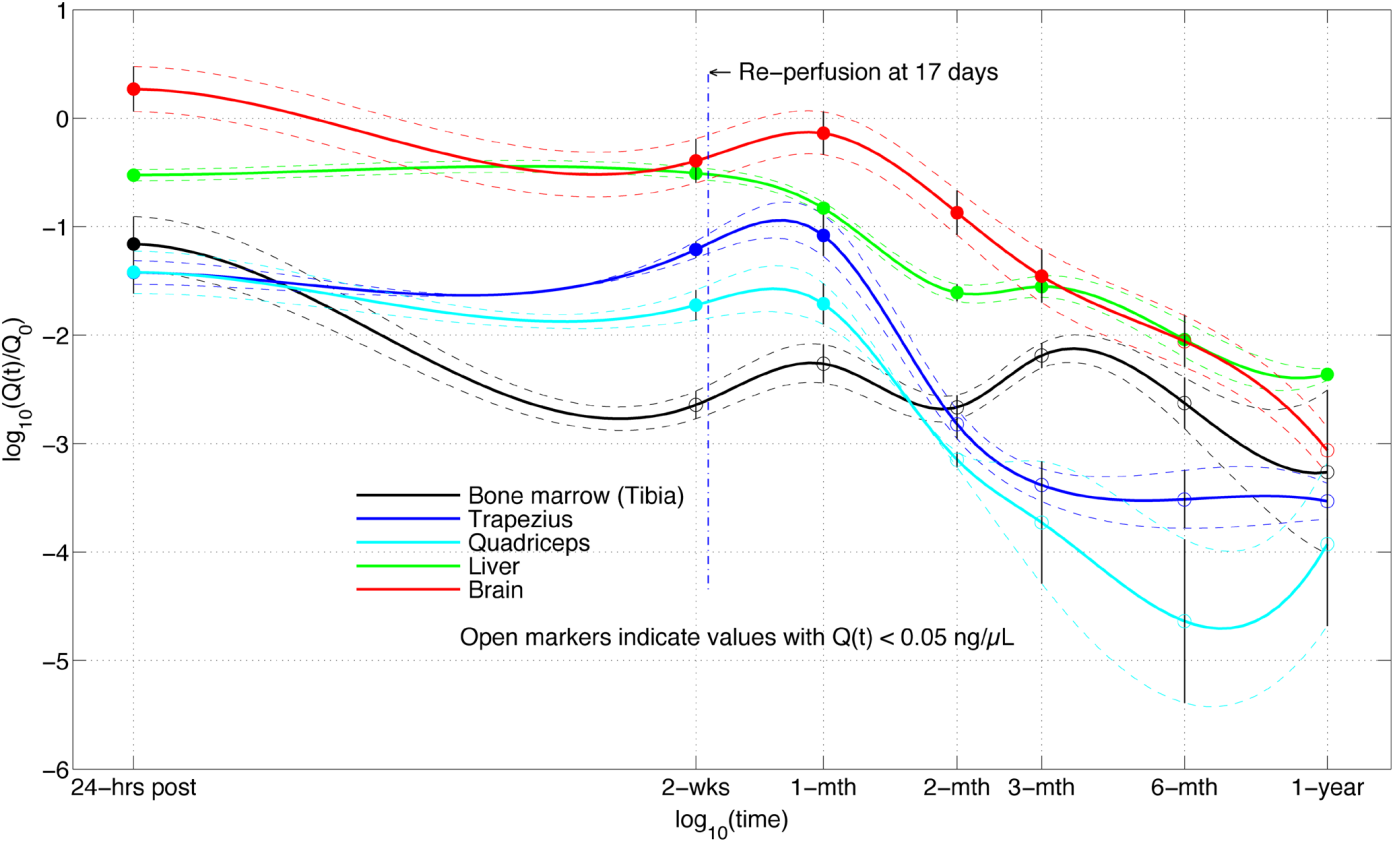
Time evolution of the relative quantification Q(t)/Q_0_ on a logarithmic scale. The dotted envelope around the estimated curve represents one standard deviation (α = 0.05). The estimated error bars assume no correlation between the sample values.

The relative amount of DNA at each location relative to its pre‐embalming level is displayed in Figure 3. Both the estimate and the relative error (α = 0.05) can be found in Table 5. Two distinct trends are observed, separated by a reperfusion event. Prior to reperfusion, each location has a characteristic saturation. Following reperfusion, the ratio consistently drops for all locations.

**TABLE 5:** For each of the seven ratios at the five locations, the ratio itself, the standard error, a point estimate of the correlation (*r*), and the probability that the correlation is non‐zero (*p*) are reported.

Sample time	24‐h post	2‐week post	1‐month post	2‐month post	3‐month post	6‐month post	1‐year post
Bone marrow (Tibia)							
Ratio	0.0692	0.0023	0.0055	0.0022	0.0065	0.0024	0.0005
Std error	0.5223	0.2940	0.4085	0.2567	0.2661	0.5433	1.7434
*r*	0.9807	−0.6896	0.9852	0.9803	−0.6285	−0.0290	−0.7546
*p*	0.8746	0.4844	0.8902	0.8733	0.4327	0.0185	0.5444
Trapezius							
Ratio	0.0378	0.0615	0.0832	0.0015	0.0004	0.0003	0.0003
Std error	0.2525	0.1762	0.4388	0.3102	0.3463	0.6147	0.3811
*r*	−0.1774	−0.6207	0.7851	0.6928	−0.3306	−0.9951	−0.9699
*p*	0.1135	0.4263	0.5748	0.4872	0.2145	0.9372	0.8434
Quadricep							
Ratio	0.0381	0.0189	0.0195	0.0007	0.0002	0.0000	0.0001
Std error	0.4496	0.3199	0.4353	0.1635	1.2966	1.7361	1.7361
*r*	−0.8460	0.1543	−0.3088	−0.9603	0.9533	−0.8633	−0.0053
*P*	0.6420	0.0986	0.1999	0.8201	0.8048	0.6633	0.0034
Liver							
Ratio	0.2990	0.3108	0.1483	0.0246	0.0280	0.0091	0.0043
Std error	0.1187	0.1214	0.1251	0.1807	0.2245	0.3744	0.1245
*r*	0.9964	0.9947	−0.9021	0.6981	−0.4074	0.5008	−0.7928
*P*	0.9456	0.9344	0.7160	0.4920	0.2671	0.3340	0.5828
Brain							
Ratio	1.8562	0.4042	0.7263	0.1344	0.0351	0.0088	0.0009
Std error	0.4782	0.4688	0.4538	0.4737	0.5602	0.5483	0.4980
*r*	−0.1236	−0.6818	0.8263	−0.9847	0.9961	0.3344	−0.9708
*p*	0.0789	0.4776	0.6191	0.8886	0.9439	0.2171	0.8458

**FIGURE 4:**
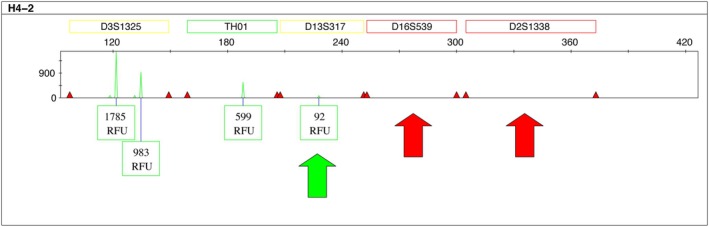
Electropherograms of 1‐year post‐embalming liver samples demonstrating allele dropout (green arrow) and locus dropout (red arrow).

Of all 120 tissue samples extracted, 75 profiles (62.5%) were generated using a DNA cut‐off value of 0.05 ng/μL, suggested by the QIAGEN DNeasy Blood & Tissue Kit [26] as well as the QIAamp DNA Blood Mini Kit [27]. For each profile, the number of alleles was counted using the pre‐embalming blood samples as the reference. Out of the 75 generated profiles, 52 (69.3%) provided complete profiles (no allele dropouts), nine (12%) had one allele dropout, eight (10.7%) had two allele dropouts, and six (8.0%) had three or more allele dropouts. Locus D2S1338 had the most significant number of dropouts, with seven profiles showing a full locus dropout and eight profiles showing a partial allele dropout. As time progressed, the quality of the DNA profiles decreased along with relative fluorescent unit (RFU) value (Figure 4). The earlier samples, such as 24‐h post‐embalming, 2‐week post‐embalming, and 1‐month post‐embalming, had good quality profiles with greater RFU values than the samples collected at subsequent time points (2‐month post‐embalming, 3‐month post‐embalming, 6‐month post‐embalming, and 1‐year post‐embalming).

**FIGURE 5:**
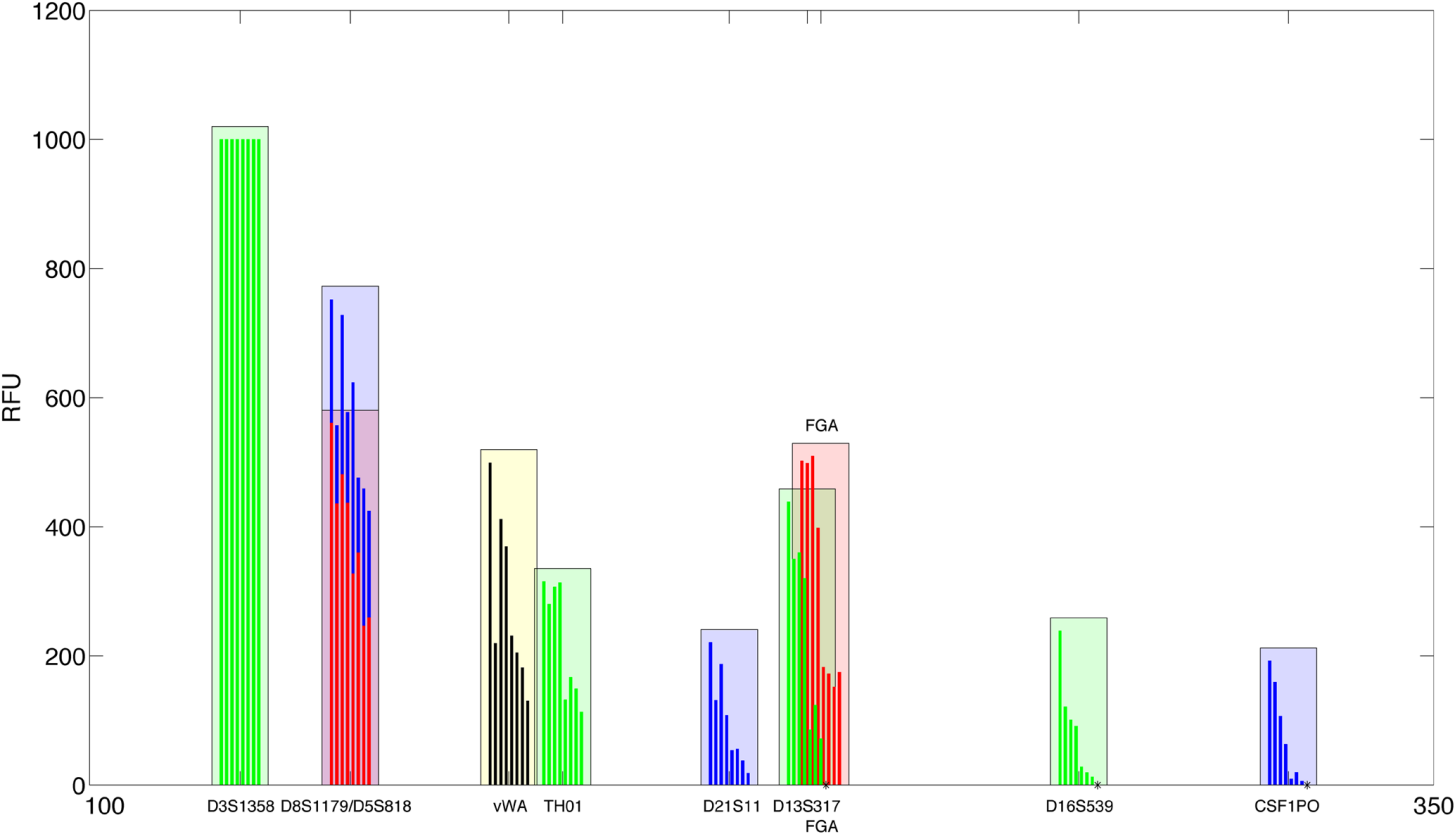
Ski slope analysis of the loci for the liver samples over the duration of the study. Collected within each bin (locus) are the samples from the eight time periods in chronological order; the far‐left sample corresponds to 24‐h pre‐embalming, and the far‐right sample corresponds to 1‐year post‐embalming. The loci are ordered in increasing amplicon size (base pairs). Data dropouts are indicated with an asterisk “*.” For each time period, the relative fluorescent unit values are normalized with respect to D3S1358.

By the 6‐month post‐embalming and 1‐year post‐embalming periods, the peak heights markedly decreased, indicating further degradation, and had more artifacts such as elevated baseline as well as more frequent allele and locus dropouts. Four of the liver samples, Figure 5 illustrates a characteristic ski slope pattern across selected STR loci spanning the full amplicon size range. In all samples, larger amplicons exhibited greater degradation, while the overall ski slope profile was preserved across all time points.

## 4 DISCUSSION

The results revealed degradation of both quantity and quality of DNA recovered from the embalmed cadaver, regardless of tissue type. While evaluating DNA degradation within embalmed tissues, this study also addressed several knowledge gaps in the current body of literature, including the examination of multiple tissue types, replication of sampling sites, and inclusion of control samples taken before embalming.

A collection of control samples, taken before embalming, established a baseline for monitoring DNA degradation. An indication of degrading DNA is the decreased quantity of DNA found within a sample, as the fragmentation and breakdown of these segments result in ineffective replication and detection. From the 24‐h pre‐embalming sample to reperfusion, all sample sites exhibited a tissue‐dependent characteristic saturation level (Figure 3). Subsequent to reperfusion, the degradation rate of this relative amount appears to be universal across tissue types.

The embalming process requires the aqueous solution to circulate the body throughout the vascular system, while being absorbed into the surrounding tissues to promote tissue fixation and preservation. As a result, tissues are exposed to a large quantity of chemicals within this time to ensure sufficient perfusion. It would be expected that the degradation of DNA is pronounced during this period when exposure is high. The brain tissue showed an increase of average DNA concentrations between 24 h pre‐embalming and 24 h post‐embalming. This may be explained by the high lipid and water content in the brain tissue, as this can cause difficulty in the weighing and separation of the brain tissue samples and/or extraction interference [28]. As the embalming solution sterilizes tissues and removes all organisms contained within tissues, contaminants present within brain tissues before embalming would have been collected during sampling and may have influenced the quantification results. Variations in sampling sites may also cause differences in concentration, as sampling locations progressively moved deeper into the tissues to reduce the chances of contamination. The tissues sampled at later dates are less exposed to the external environment, which may have resulted in less degradation of DNA compared to the surface layers sampled during the pre‐embalming sampling period.

Within the scope of this project, DNA yield and STR success variability were implemented by sampling three replicates from five different tissue types: quadriceps (rectus femoris), bone marrow (tibia), brain (parietal lobe), liver, and trapezius (middle portion) at eight designated sampling intervals, which were extracted, quantified, and amplified to compare DNA yield and the ability to generate an interpretable DNA profile. In this study, on average, the liver samples contained statistically higher quantities of DNA, whereas bone marrow (tibia) samples, on average, contained statistically lower quantities of DNA. This is the opposite of findings from research such as Wheeler et al. [12]. The variation in methodologies may have contributed to the difference in DNA concentration. More specifically, Wheeler [12] sampled the embalmed cadavers at one‐time point, lacking repeated exposure to the external environment, which had occurred during each sampling time point within this research. This exposure could have influenced various tissues differently, such that it promoted the decomposition or contamination of samples at some sample sites more than others.

There was no statistical difference between the average concentration of DNA obtained from the tissue sampled nearest the perfusion site (quadriceps) and that sampled farthest from the perfusion site (trapezius), indicating thorough and even perfusion throughout all tissue types, independent of location. Similarly, in Wheeler [12], while it was found that sampling locations of the buttocks on average resulted in increased concentrations of DNA than the trapezius, these values were not statistically different.

Our results demonstrate a decrease in STR peak heights and an increase in allelic dropout as the time since embalming increased, indicating a decline in DNA quality. This finding supports the published literature, which indicates that DNA will continue to degrade over time, as evidenced by the decrease in both the quality and quantity of a DNA profile, as depicted in the RFU values (Figure 5) [29]. In the research done by Czado [30], most of the profiles generated had 33.5% of expected alleles present, which did not significantly change after implementing methods to repair the damaged DNA template. Conversely, in this research, 69.3% of profiles retained all expected alleles. The STR success of the liver samples in this research may be attributed to the shorter embalming period (up to 365 days post‐embalming) compared to the 814‐day embalming period for some of the Czado [30] samples. STR profiling in this study was employed as a qualitative indicator of DNA degradation. The results demonstrated fragment length–dependent amplification success, with shorter STR amplicons consistently showing higher recovery than longer alleles. This pattern is characteristic of the well‐described ski slope effect observed in degraded DNA samples [31, 32] and represented here for the liver in Figure 5.

The bone marrow (tibia) samples did not produce DNA profiles after 24 h post‐embalming and were the least reliable in generating a profile. For the purpose of sampling within this study, aside from sampling site efficiency, the most effective method for obtaining the required amount of tissue sample also needed to be considered. This is not something that is typically considered when taking a biopsy sample, as the often times a needle is used to obtain a small amount of tissue. In a study by Fakhri [33], bone marrow from common sampling sites (i.e., femur, pelvis, and humerus) were extracted for the purpose of developing an effective protocol for continuous bone marrow extraction from human cadavers [33]. In selecting the optimal bone marrow sampling site for this study, care was taken to minimize peripheral blood contamination to ensure that the DNA originated from bone marrow rather than blood [34]. This was not taken into consideration in the study by Fakhri [33] since the sampling of bone marrow was conducted on decomposing cadavers, and blood was not a concern. The Fakhri study designated the pelvis as the preferred area for biopsy samples, as the area is easily penetrated by the needle [33]. While a needle biopsy is a common method of sampling bone marrow, as it bypasses the muscle and fat tissues, it only collects a small specimen (c. 20 mm), ruling it out as a viable collection device for this research [33]. To allow for the collection of larger amounts of bone marrow, it was required to cut into the bone and extract the bone marrow manually. This deemed the pelvis unsuitable as it is a difficult area to sample by hand.

The femur was also considered as a potential sampling site for bone marrow, given it is commonly used as a marrow source for DNA analysis, as the femur contains a high density of compact cortical bone which protects DNA from environmental degradation and it is easily accessible for manual extraction [21, 35]. Ultimately, the femur was excluded as the bone marrow sampling site due to the high risk of contamination with peripheral blood during sampling conducted before the embalming process. The tibia was therefore designated as the collection site for the bone marrow samples based on the same criteria used for evaluating the femur; it is easily accessible and presents a lower risk of blood contamination. When sampling for casework, bone marrow from the femur and pelvis can be used, as there are no restrictions, such as utilizing blood as a pre‐embalming control.

Similar to the bone marrow (tibia), the trapezius and quadriceps muscle samples were only slightly more reliable, failing to produce DNA profiles after 1 month post‐embalming. While the trapezius and quadriceps muscle tissues both produced a DNA profile beyond the 24‐h mark, the bone marrow, trapezius, and quadriceps muscle tissues were not suitable sources for long‐term DNA sampling after embalming. The liver samples were the most robust and yielded DNA profiles up to 1 year. Although more allelic dropout was observed in the liver samples at the later sampling times, the liver remained more reliable than the bone marrow, trapezius, and quadriceps samples.

This research was meant to track the degradation of DNA within formalin‐fixed tissues over time, something novel to the forensic science community, as no similar published research exists. Although it is challenging to compare findings due to the limited number of previous publications, these encouraging results suggest a promising direction for uncovering consistent trends in the DNA degradation rates.

A number of limitations should be acknowledged. First, the sample size was limited to a single cadaveric donor. Although the aim of this study was to investigate DNA degradation across different tissue types, reliance on one donor restricts the ability to assess intra‐individual variability. Second, DNA yield from some sample types averaged below the threshold required for successful amplification. As a result, these samples could not be amplified and did not yield STR profiles.

To address the limitations of this study, future work should focus on sample types with DNA quantities below the amplification cutoff threshold, as many forensic laboratories would concentrate such samples prior to analysis. Exploring the relationship between embalming solution and DNA degradation with simulated real‐world conditions would also be beneficial. Due to the required storage conditions and the study duration, the cadaver underwent two perfusions to prevent the loss of solution and tissue decomposition. This may not always occur in real‐world embalming, depending on the time elapsed between death and interment. This research focuses on tracking the degradation pattern of DNA, rather than emulating current internment procedures. Implementing a different extraction method, as used by Snedeker et al. [36] resulted in obtaining quantifiable and amplifiable DNA from embalmed samples. Afrifah et al. [37] describes another extraction method, which was able to yield detectable DNA quantities from embalmed tissues (brain, cartilage, and bone) and complete STR profiles with varying peak heights. To determine their influence on DNA degradation and to establish a greater understanding of how the individual components within embalming solutions promote the degradation of DNA, studies could be conducted that focus on isolating the individual ingredients in embalming solutions.

## 5 CONCLUSION

The objectives of this research were to evaluate DNA degradation in several tissue types sampled from the embalmed cadaver by establishing planned and controlled time intervals for sampling and using controls pre‐embalming. The effects of embalming on different types of tissue were assessed to determine the most reliable sampling location for DNA extraction up to 1‐year postmortem. It was determined that there was no significant difference between tissue types in relevance to the distance from the perfusion site. However, there was a substantial decrease in DNA quantity and quality of DNA profiles throughout the different tissue types over time. The degradation rate exhibited a consistent profile, independent of the tissue type.

Furthermore, before reperfusion, the degradation rate was minimal, and after reperfusion, the decay rate of the relative DNA amount exhibited a consistent pattern across all tissues. The liver was the most efficient sampling site, yielding the largest amount of DNA before embalming and retaining viable DNA for up to 1‐year postembalming. The least favorable sampling site, yielding decreased concentrations and insufficient quantities of DNA, was the bone marrow (tibia). DNA profiles were not present at this location past the 24‐h postembalming sample. There was no significant difference in the quantity of DNA recovered from the “near perfusion site” quadriceps tissue compared to the “away from perfusion site” trapezius tissue. Interpretable STR profiles were obtained from postmortem DNA samples collected in the trapezius, quadriceps, liver, and brain tissues for up to one year post‐embalming. Ski slope analysis showed predictable degradation for viable samples. This information can be used to determine the most efficient sampling method and identify ideal sampling sites that retain DNA for more extended periods after embalming, thereby yielding a reliable STR profile for the identification of human remains. When a DNA profile is required to identify human remains, the liver is the preferred tissue to consider due to its ability to generate a DNA profile.

## CONFLICT OF INTEREST STATEMENT

The authors declare that they have no conflicts of interest relevant to this manuscript.

## Supporting information


Appendix S1.

## Data Availability

The data that support the findings of this study are available on request from the corresponding author. The data are not publicly available due to privacy or ethical restrictions.
