# Supplementary material for: A 1‐year forensic evaluation of DNA degradation and STR typing in embalmed human tissues: Muscle, brain, liver, and bone marrow
Source: J Forensic Sci. 2026 Apr 24;71(4):1829–40. doi: 10.1111/1556-4029.70345 (PMC13340956; doi:10.1111/1556-4029.70345)
Supplement: Supplementary file 1 — Appendix S1. [file JFO-71-1829-s001.docx]

**Appendix A**

**Locations Sampled at Each of the Eight-Time Points and Corresponding Sampling Methods**

| **Sampling Locations** | **Sampling Method** |
| --- | --- |
| Bone Marrow (Tibia) | A longitudinal cutaneous incision (~15 cm) was made to expose the right tibia. Osteotomy was performed to create 2cm x 5cm window access to the bone medullary cavity and sample the bone marrow |
| Trapezius muscle | Medial-to-lateral cutaneous incisions were made within the superficial fascia to gain access to both the left and right trapezius  For representative sampling, three samples were collected from the left and two from the right and this alternated at each sampling time |
| Quadriceps (Rectus Femoris muscle) | A longitudinal cutaneous incision was performed on the anterior surface of the right thigh to expose underlying muscle tissue |
| Liver | Subcostal laparotomic incision to achieve access to the liver |
| Brain (Cerebral cortex) | Lateral incision posterior to the coronal suture and skull cap peeled back. An approximate 4cm x 5cm window was cut into the skull using a bone saw, which was removed to sample the brain (parietal lobe) |
